# Family dominant hypothesis for the effect of family of origin on the mental health of offspring: evidence, mechanism, and implications

**DOI:** 10.3389/fpsyt.2026.1733077

**Published:** 2026-01-28

**Authors:** Zongpei Dai, Yutong Luo, Jieying Tan, Li Hu, Qin Dai

**Affiliations:** 1 Research Center for Children’s Brain and Cognitive Sciences, Chongqing University of Education, Chongqing, China; 2 Department of Pain, The Southwest Hospital, Army Medical University, Chong qing, China; 3 Key Laboratory of Mental Health, Institute of Psychology, Chinese Academy of Sciences (CAS), Beijing, China

**Keywords:** family dominant hypothesis, family of origin, intervention, mechanism, mental health of offspring

## Abstract

Individuals suffering from mental illness often report an abnormal family of origin. Previous family theories have primarily focused solely on family risk factors or treated family members equally and have largely overlooked the critical role of family of origin in offspring development. In this study, we proposed a *Family Dominant Hypothesis* to emphasize the critical role of family of origin in the lifelong mental health of offspring. The core concept is that family and environmental variables, encompassing both risk and protective factors, contribute to different mental health outcomes in offspring, with the family of origin playing a directly dominant and indirectly mediating role in child development. This theoretical hypothesis highlights the dominant and mediating role of family of origin, considering both risk and protective factors--including biological genetics, family conditions, parental mental wellness, the relationship between parents, parenting style, and the parent-child relationship--in child development. Potential neurobiological mechanisms underlying the influence of the family of origin were also explored. Meanwhile, potential family interventions targeting the identified risk factors were proposed. The Family Dominant Theory is proposed to draw attention from society, particularly families and young parents, to emphasize the importance to family of origin environment. A comprehensive understanding of family of origin can help establish a healthier family environment and promote lifelong mental health of offspring.

## 1 Introduction

It is well known that childhood happiness guarantees a better lifetime life-satisfaction, while childhood adversity functions in a contrary way (1). Psychoanalysts have long emphasized the role of early-life adversities in the development of mental disorders (2). Early-life adversity comprises multiple dimensions (3), including individual-related issue (personality, temperament, emotionality, cognitive style, et al.) (4), family-related issue (parental relationship, parenting style, et al.) (5, 6), school-related issue (teacher-student relationship, learning achievement, et al.) (7), and society-related issue (peer relationship, neighborhood conflicts, et al.) (8). However, the relative impact of these dimensions on the mental health of offspring has not yet been established. Importantly, an increasing number of studies have focused on family-related adversities (9, 10). However, the role of family of origin in offspring’s mental health has not been systematically examined in the literature.

Generally, individuals have two families. The first is the family of origin, in which the individual grows up. The second is the family of procreation, which is influenced by the family of origin and formed in adulthood. Bowen’s *Family System Theory* emphasizes the importance of family as a system (11). All family members are embedded within this system, and mental distress arises from interactions among members rather than from isolated development. According to this theory, internal interactions among family members can affect overall family functioning and have profound and lasting effects on the mental health of family members. The theory focuses on the differentiation of individuals, aiming to empower each member of the family-husbands, wives, parents, children and others - equally. However, the effects of the family system may differ across members due to their distinct roles, with children being uniquely influenced compared with parents. For a child, the family of origin is the primary context in which they are raised, and it is crucial for the development of psychological adaptive capacities. For couples, the family of procreation reflects characteristics of the family of origin. As noted in Toxic Parents, “Our parents plant mental and emotional seeds in us -seeds that grow as we do. In some families, these are seeds of love, respect, and independence. But in many others, they are seeds of fear, obligation, or guilt” (12). This warrants the development of a theory to focus on the family of origin and to address children’s mental health from this perspective.

## 2 Proposal of family dominant hypothesis

We thus propose a family dominant hypothesis to comprehensively examine the direct and mediation roles of the family of origin in the mental health of offspring, considering both risk and protective perspectives.

### 2.1 Description of family dominant hypothesis

**Figure 1:**
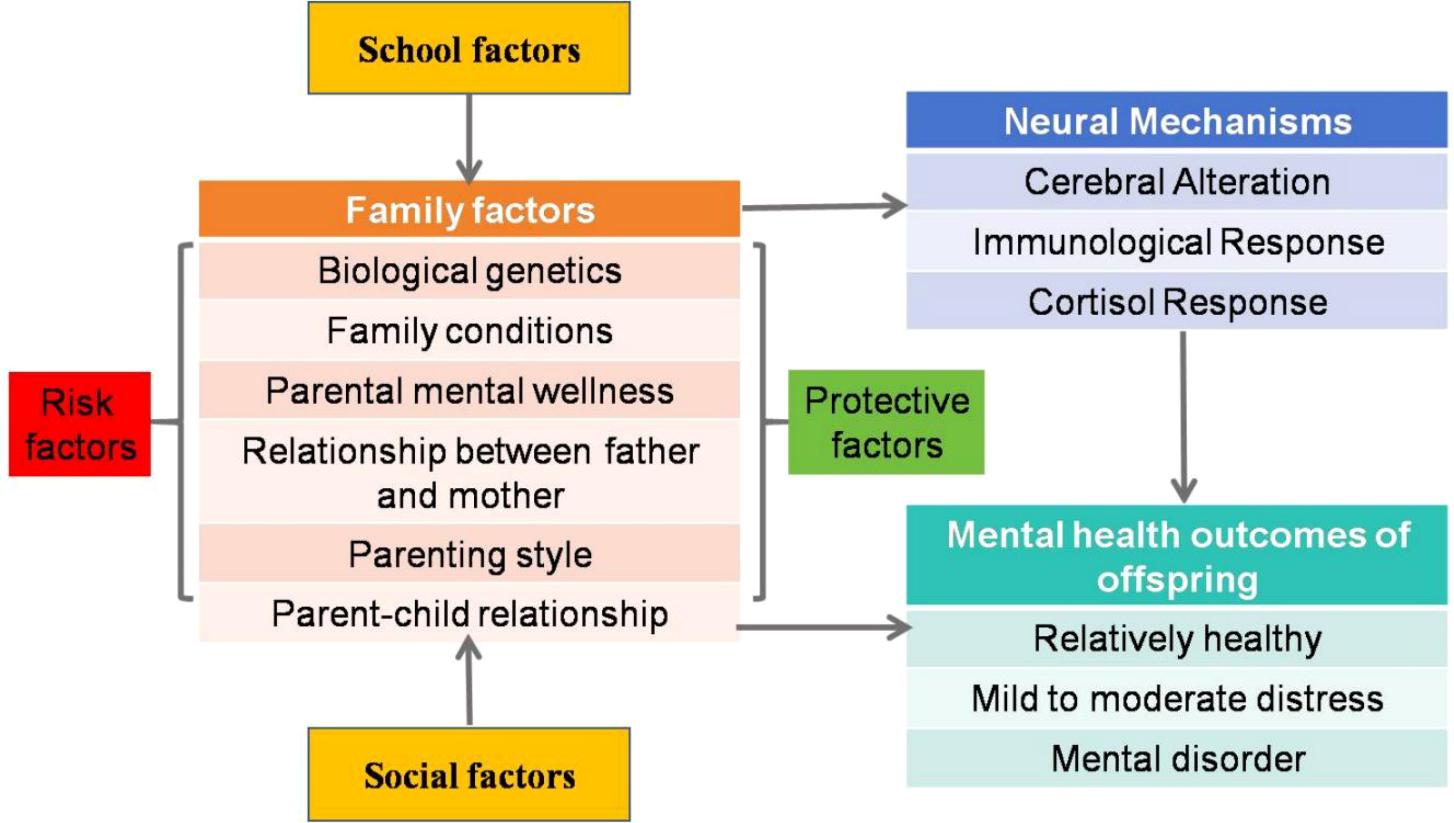
Family dominant hypothesis for the mental health of offspring.

By integrating multi-factors involved in early-life adversity (4, 6–8) with the Family Stress Model (13) and the Risky Family Model (14) and Family System Theory (11), we propose a Family Dominant Hypothesis to illustrate the crucial role of the family of origin in offspring mental health outcomes. In this theoretical framework, we hypothesize that the family of origin had an early and unique impact on offspring mental health outcomes (15). Second, offspring mental health is influenced by the combined effects of risk (16) and protective (17) family of origin factors. Third, family of origin variables may mediate the influence of school (7) - social (8) factors on offspring mental health outcomes (18). See **Figure 1**.

### 2.2 Main points of family dominant hypothesis

#### 2.2.1 Early and unique impact of family of origin on mental health of offspring

The family of origin possesses theoretical primacy as the first-order explanatory system. According to *Ecological Systems Theory* (19), the family of origin is the earliest, most intimate, and longest-lasting relational context that children encounter, which is an irreplaceable starting point environmental system for explaining children’s mental health. This means that family of origin holds developmental temporal priority, which exerts influence earlier than other ecological systems. Within this context, children gradually develop stable patterns. These experiences directly shape their functioning in subsequent developmental systems, determines the initial psychological resources available to them and their readiness to enter other contexts. Children form core psychological infrastructure within the family of origin, which guides how they function in subsequent systems, for example peer and romantic relationships (20). Moreover, previous research found that multiple family adversities substantially increase the risk of major depressive disorder in adulthood (15). Consistently, stressful experiences in the family of origin during childhood contribute to the development of mental disorders during adolescence and persist into young adulthood (21). These findings suggest that the family of origin has a direct and long-lasting influence on offspring’s mental health across the lifespan. These underscore the dominant role of the family of origin on offspring mental health outcome, ranging from relatively healthy to mild/moderate distress to mental disorder (15).

Notably, previous research on early-life adversity has over-represented the scope of early-family adversity, often extending beyond the family domain to varying degrees. This indicates that family of origin is the core environment for an individual’s development, further underscores the dominant role of family of origin. For example, Sorocco et al. (16) assessed early-life adversity through retrospective reports, including low socioeconomic status and personal experience of physical or sexual abuse and/or parents separation before age 16,with family adversity constituting a significant part. Similarly, in the three-hit concept of vulnerability and resilience to stress-related mental disorders, hit-2 - the early-life environment - comprised 15 elements, 9 of which belonged to the family dimension (5). Some studies even considered early family adversity equivalent to early-life adversity. For example, Korkeila et al. (22) used a brief 6-item scale measuring family adversity to assess early-life adversities. Consistently, several tools originally designed to assess early family adversity have been used as proxies for overall early-life adversity. These measures encompass a broader scope than early family adversity, such as the ‘enriched’ family adversity index (23), the Alcohol Use Disorder and Associated Disabilities Interview Schedule-IV (AUDADIS-IV) (24), and the Risky Families Questionnaire (14). The over-representation of these tools suggested that researchers implicitly consider early family adversity as the primary component of early-life adversity, indirectly supporting the dominant role of the family of origin in offspring development. Based on the above discussion, it can be concluded that although multiple dimensions (individual, family, school, and social) contribute to early-life adversity, the family of origin plays the dominant role in children’s mental health.

#### 2.2.2 Combined effect of risk and protective family factors on mental health of offspring

##### 2.2.2.1 Buffering interaction between risk and protective factors

Factors within family of origin can be categorized into two aspects: risk (16) and protective (17) factors, which may interact in a buffering manner, jointly influencing offspring well-being. On the one hand, risk factors might attenuate the benefits of protective factors. For example, evidence showed that the benefits of having a married mother for children’s emotional support were weakened by an adverse family environment during upbringing (25). Conversely, protective factors may buffer the adverse effects of risk factors. Research on Intimate Partner Violence (IPV) reported that children who had contact with a less- or non-violent father could buffer the impact of IPV on their externalizing problems (26). Similarly, mother-child cohesiveness moderated the relationship between the persistence of domestic violence and children’s anger (27). These pieces of evidence support the buffering interactions between risk and protective family factors in determining offspring’s mental health outcomes.

##### 2.2.2.2 Decisive accumulation from risk and protective factors

In addition to buffering interactions, either risk or protective factors may exert a decisive effect on children’s mental health, since that some effects may be mutually buffered, while remained effects may still manifest. Wille et al. (28) reported that the presence of more risk factors in a family was associated with a higher prevalence of mental health problems in offspring. In contrast, substantial resources at the individual, family and social levels were associated with a reduced occurrence of mental health problems, particularly when children lived in families with fewer risk factors (28). In other words, the cumulative effects of both risk and protective family factors may determine which side predominates. In general, alert awareness and initiating action occur only after the harm has occurred (29), which takes a lot of time and energy to repair and rebuild. Thus, it is crucial to identify and foster appropriate protective factors before negative outcomes arise, especially in families with more risk factors.

#### 2.2.3 Mediation of family of origin between environment and the mental health outcome of offspring

The dominant role of the family of origin is further manifested in its causal mediation centrality. Family of origin is the core but not the only isolated place for one’s growth. According to Ecological Systems Theory (19), the family of origin constitutes the most proximal micro-system and is therefore the earliest system to exert a mediating function. The impact of external stressors on children’s mental health is primarily filtered through the family processes (3, 11). Ultimately, these experiences are consolidated into the child’s internal working model (30), contributing to different mental health outcomes. That is, children gain experiences from the external environment, and the family helps them process these experiences in ways that may heal, harm, or strengthen one’s sense of self. Thus, the leading role of the family of origin is manifested not only directly but also indirectly.

Importantly, previous empirical studies have provided obvious evidence. For example, children who received stable parenting were more likely to recover from the psychological stress of community violence than their counterparts (31). Similarly, family relationship (the level of cohesion, conflict, and expressiveness) served as mediating factor in the impact of school bullying involvement on adolescents’ depression (32). Moreover, parental support mediated the association between youth life-stress and suicidal ideation (33). These findings suggest that the family of origin mediates the effects of external environment on the mental health of offspring. Indeed, factors from the external environments may alter the degree of influence exerted by the family of origin, ultimately resulting in either better or worse outcomes for children.

### 2.3 Differences between the family dominant hypothesis and previous theories

Bronfenbrenner’s Ecological Systems Theory (19) emphasizes that individuals are nested within a series of interacting environmental systems, and the systems interact with individuals and influence their development. In this theory, although micro-systems (such as family and school) are located at the innermost layer of the entire ecosystem, however, various systems are emphasized, all have significant impact on individuals, none of which is overvalued or undervalued. Moreover, variety of family systems is represented in this theory instead of unique family of origin. Besides, this theory applies to general population in a broader framework other than specific roles such as offspring. Family Dominant Hypothesis posits a hierarchical structure where the family of origin is not merely parallel to other systems but functions as the foundational driver. Notably, family of origin has the temporal priority in the development process of children (34), which makes it necessary to place family of origin a primary position in influencing offspring’s development. It highlights the dominant impact of the family of origin on offspring’s mental health, which is not addressed in Ecological System Theory.

Bowen’s Family System Theory (11) conceptualizes the family as a system in which individual distress arises from patterns of interaction within the system rather than from isolated personal dysfunction. It aims to empower individuals (husband, wife, parents, children, et al.) in a family system equally. Notably, different kinds of families are included in this theory instead of unique family of origin. Family Dominant Hypothesis focuses on unique family of origin, recognizes that child reoccupy a subordinate and developmentally dependent position in the family structure. Consequently, rather than treating all members as equal agents, the Family Dominant Hypothesis focuses on how the family of origin exerts a predominant influence on the mental health trajectories of offspring. It lays the basic framework for children’s experiences, interpretations and adaptations in other environments (e.g., school, social context).

Family Stress Model (13) suggests that family stressors can undermine parents’ mental health, causing preoccupation with their own problems and, consequently engage in inappropriate parenting practices (e.g., harsher discipline). However, this model primarily emphasizes solely on the role of family economic stress in children’s development and conceptualizes key family variables (e.g., parental psychological distress and parenting practices) mainly as mediating mechanisms. It does not systematically delineate the early and unique effects of family factors on offspring mental health, nor does it sufficiently integrate broader environmental influences. Besides, it does not discuss crucial protective variables of family function that may buffer or offset the adverse effects of stressors. In contrast, Family Dominant Hypothesis moves beyond an exclusive focus on family strain to integrate family risk and protective factors for elaborating the dominant and direct influence of family of origin on offspring’s mental health outcomes, and highlights interaction between family stress and environmental stressors (e.g., school or social contexts).

Similarly, the Risky Family Model (14) demonstrates that early exposure to adverse family environments disrupts emotional processing and physiological regulation, which in turn impair psychosocial functioning and ultimately physical health. While this model emphasizes the link between risky family exposure and psycho-physiological outcomes, it does not account for how family risk may mediate the influence of environmental (school or social) stressors on children’s development, nor does it explicitly incorporate potential protective variables in family of origin. On the contrary, the *Family Dominant Hypothesis* elaborately discusses the risk and protective factors within the family of origin and highlights the relationship between these factors and the external environment.

Family of origin is not only a critical source of early experiences for children, but the primary system for organizing how children enter, understand and adapt to all other developmental situations. However, none of the existing theories explicitly acknowledge the leading and mediating role of the family of origin in shaping offspring development, nor do they systematically address the risk and protective factors of the family environment. The Family Dominant Hypothesis addresses these gaps by proposing a clearer and more systematical theoretical proposition, it makes three key contributions: (1) It conceptualizes the family of origin as holding a developmentally early and unique effect- in determining offspring mental health outcomes, highlighting the need for young parents and clinicians to intervene early to mitigate family risk factors to guarantee better outcomes of offspring. (2) It grants equal importance to risk and protective family factors of family of origin, recognizing their potential to shape offspring’s mental outcomes. (3) It integrates environmental (school and social) variables, emphasizing their indirect effects on mental health through the mediating function of family of origin. Together, these contributions articulate a unified theoretical account of why the family of origin holds a dominant role in offspring mental health. Evidence supporting these propositions is listed in the following section.

## 3 Evidence for risk and protective family factors in offspring mental health outcomes

**Figure 2:**
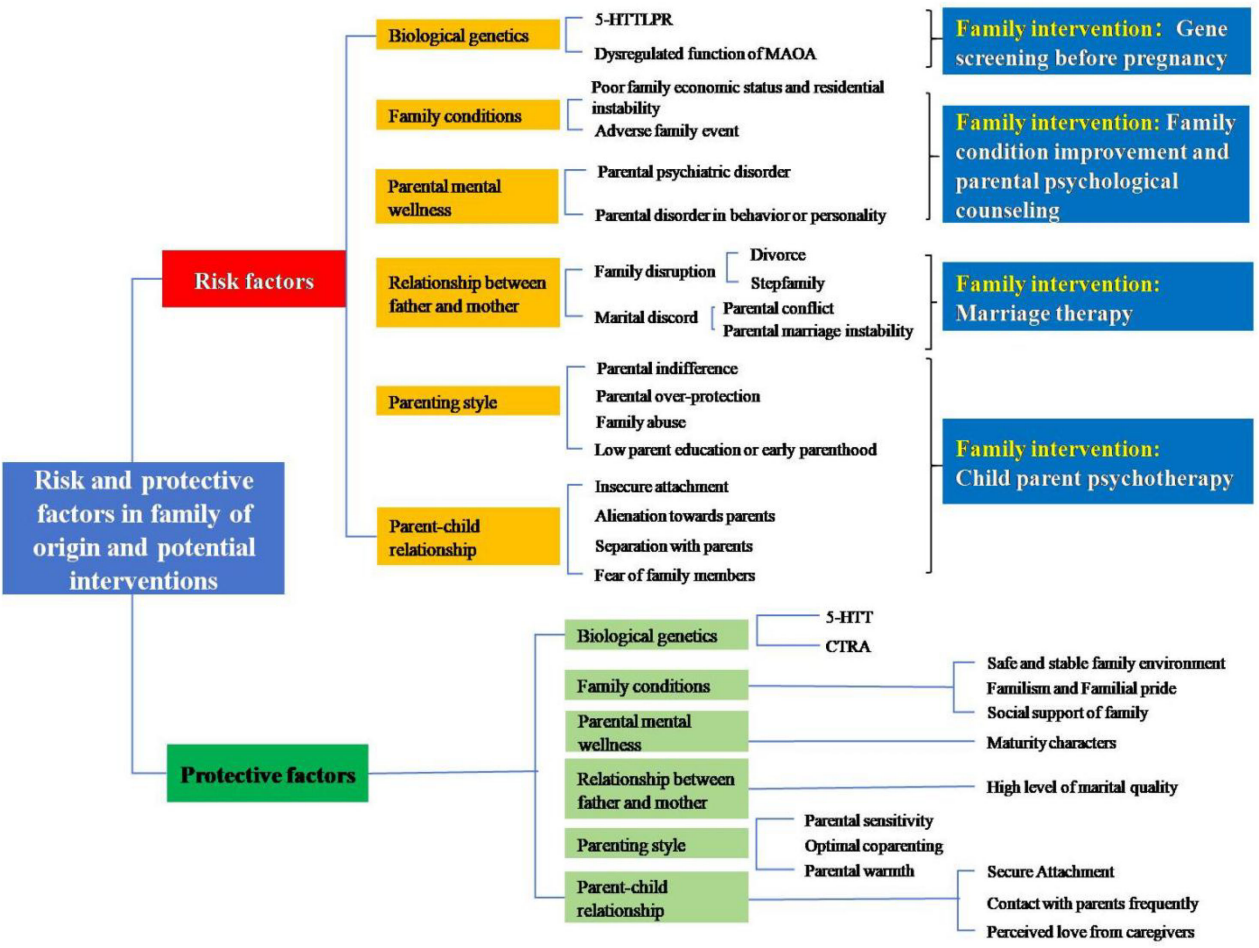
Risk and protective factors in family of origin and potential interventions.

Given the complexity of environmental variables and majority family risk literature (13, 14) in the early-life adversity literature, this section systematically examines six key family dimensions from both risk and protective perspectives: biological genetics, family conditions, parental mental wellness, relationship between father and mother, parenting style, and parent-child relationship. See **Figure 2** and **Supplementary Materials (Table S1)**.

### 3.1 Risk family factors in offspring mental health

#### 3.1.1 Biological genetics

Specific genetic predispositions can increase an individual’s risk for mental disorders, particularly when early-life adversities are present (35). Meta-analyses have confirmed significant interactions between the serotonin transporter gene polymorphism (5-HTTLPR) and stress (36) or early-life adversity (37) in predicting depression. Specifically, s-allele carriers with stressful life events have increased odds of developing depressive symptoms (38). Beyond 5-HTTLPR, meta-analysis indicate that common regulatory variations in monoamine oxidase A (MAOA) exert a moderate effect on male antisocial behavior in the context of childhood maltreatment (39). Male carriers of the MAOA-L genotype who experienced maltreatment were more likely to exhibit antisocial behavior than their MAOA-H counterparts (40). These findings suggest that 5-HTTLPR, dysregulated function of MAOA are risk factors for adverse mental health outcomes. It is clear that interactions between biological genetics and family environments play a critical role - a topic further discussed in the following sections.

#### 3.1.2 Family conditions

##### 3.1.2.1 Poor family economic status and residential instability

Family conditions refer to a constant physical and psychological environment in which children grow up. The most representative stuff might be family economic status. Family poverty has been widely recognized as a significant risk factor, particularly linked to offspring’s mood disorders (6, 41), psychological distress (42), and behavioral disorders (43). It has also been associated with a higher external locus of control (44) and an increased risk of suicide among children (45). Moreover, low parental socioeconomic status and high residential instability have been related to elevated lifetime risks of depression, greater recurrence, and a reduced likelihood of remission (46). Adverse family event. In addition, children raised in families that experienced a high level of adverse life-events were more likely to develop lifetime depression (47). Likewise, adverse health events in childhood and early adolescence were significant predictors of late-adolescent attention-deficit/hyperactivity disorder (ADHD) (48). In conclusion, poor family economic status, residential instability, and frequent adverse family events are risk factors.

#### 3.1.3 Parental mental wellness

##### 3.1.3.1 Parental psychiatric disorder

Family studies had consistently shown that first-degree relatives of bipolar patients were at increased risk for mood and anxiety disorders compared to those without such family histories (49). In particular, a history of parental psychiatric disorder strongly predicts the occurrence of mood disorders in offspring (6). Similarly, a family history of dysthymic disorder had been linked to higher levels of offspring’s depression (50) and greater likelihood of ADHD symptoms (51).

From a gender perspective, maternal mental disorders have been repeatedly associated with negative mental outcomes in children, including depression and psychological distress (42), chronic disabling fatigue (52), and higher risk in reduction of cerebral activity (53). Maternal early-life stress can alter cortisol responses to an ecologically relevant postpartum stressor, and this dysregulation was often mirrored in offspring (54). Both prenatal and postnatal maternal mood disturbances exerting impacts: prenatal depression and anxiety is associated with poor socio-emotional development in offspring (55), while postnatal depression increased the risk of offspring depression by the age of 16 (56). Longitudinal evidence spanning 81 months further demonstrated that maternal depression had increased the odds of child’s high emotionality (57). While the influence of paternal mental health had been less studied, available evidence suggests similar patterns. Paternal psychological distress had been mildly but positively linked to behavioral and emotional difficulties in children (58). A recent cross-lagged analysis also reported a reciprocal association between paternal psychological distress and subsequent child emotional symptoms across ages 3 to 14 (59).

In general, the influence of parents’ psychiatric disorders on mental health of offspring may be influenced by family genes. But studies of adoptive families have found that emotional instability in adoptive parents can also affect a child’s mental well-being (60). Therefore, the impact of parental mental wellness on offspring’s well-being requires consideration of both genetic and environmental issues.

##### 3.1.3.2 Parental disorder in behavior or personality

Beyond the direct impact of parental psycho status, parents’ behavioral patterns also play a crucial role in shaping offspring’s psychological development. Evidence indicated that maternal conduct disorder was a stronger signal of “pre-schizophrenic” behavior in children, whereas paternal conduct disorder was a greater signal of externalizing behaviors (61). Family history of addiction was independently associated with offspring’s poor emotion regulation and negative moods (16). Specifically, frequency of alcohol consumption, drunkenness, and smoking were associated with offspring’s temperament and character dimensions in their early adulthood (62). Higher scores on harm avoidance among mothers and lower scores on self-directedness among fathers, were associated with an increased risk of suicide attempts among adolescents (63). In parents with autism spectrum disorder, self-directedness emerged as a potential autistic trait in offspring (64).

To sum up, parental psychiatric, behavioral, and personality disorders are risk factors.

#### 3.1.4 Relationship between father and mother

##### 3.1.4.1 Family disruption

Studies have shown the effects of family disruptions on offspring. It was reported that family dissolution increased the likelihood of mood disorders (6), suicidal ideation (45), and the lifetime risk of depression (46) in offspring. Importantly, parental divorce had a long-term influence on the early antecedents of depression and antisocial behavior in adulthood (65). An early experience of family disruption was significantly associated with delinquent behavior at age 11 (66). Children who spent equal time with both parents after parental divorce might find life easier (67), while not living with one of the parents was associated with poorer outcomes (68). The possible reason was that a divorced family not only implied the “loss” of the family itself but also the loss of opportunities to interact with one of the parents (69). Moreover, parental divorce was associated with lower relationship commitment and confidence, particularly for women, which increased the potential risk for their own marital development (70, 71). Moreover, individuals who experienced parents’ relationship instability before age 5 were more likely to report a history of sexual partnerships at age 16 or an episode of major depression during adolescence (72).

Some divorced people eventually remarry, and these remarriages often involve children from previous unions, thus forming stepfamilies. Compared to children in single-parent families, those who live in stepfamilies are more likely to experience unintentional injuries, which was attributed to higher exposure to psychosocial risks (73). However, the negative impact of stepfamilies on offspring has not been widely acknowledged. For example, Nicholson et al. (74) noted that although young people living in a stepfamily had increased risks of poor psychosocial outcomes, many of these associations arose from confounding social, contextual, and individual factors rather than the stepfamily per se. Therefore, the invisible harm might be partially present in the family of origin and subsequently manifest in the stepfamily.

##### 3.1.4.2 Marital discord

Living in a 2-parent household has no guarantee of good child outcomes. The happiness of offspring after parental divorce decreases significantly when they live with a low-conflict parent, whereas it is even lower for those who remain with a high-conflict parent in an intact marriage (75). Parental conflict resulted in deleterious consequences for children’s mood, health, and behavior (76–78). Highly troubled marriages have significant and sometimes underappreciated negative effects on children’s adjustment (79). This conclusion was confirmed by later studies, in that high levels of depression in children were associated with disturbed family relationships (42, 80). Constant parental conflict increases negative emotions such as depression and anger due to an accumulation of insecurity (81).

In summary, family disruption and marital discord were regarded as risk factors for children’s mental health.

#### 3.1.5 Parenting style

Parenting style is not a new topic in child development. Early experiences with caregivers shape children’s performance in developmental tasks, which in turn contribute to psychosocial outcomes (82, 83).

##### 3.1.5.1 Parental indifference

Among different types of parenting, parental indifference has been identified as a risk factor for children’s development, which increased the risk for both depression and anxiety in adulthood (84). In addition, low parental care is associated with increased risk of abuse by a biologically unrelated perpetrator before the age of 11, which makes an independent contribution to the affective symptoms (85).

##### 3.1.5.2 Parental over-protection

In females, higher levels of sociotropy were related to greater maternal protection (86). Indeed, perceived parental overprotection was significantly related to heart-focused anxiety (87), dysfunctional attitudes about achievement and dependency (88), functional somatic symptoms (89), as well as sleep disturbance in adulthood (90). Similarly, the perceptions of ‘not having a controlling mother’,’ having a caring father’, and ‘not having controlling parents’, were associated with a lower risk of mental illness (91).

##### 3.1.5.3 Family abuse

Exposure to victimization in early-life family environment has been associated with a wide range of mental health problems, such as depression, anxiety, post-traumatic stress disorder (PTSD) (9, 92, 93), and antisocial behavior in adulthood (65). Childhood physical and sexual abuse was associated with suicide attempts (94). Maltreated children were more likely to describe their early parental bonding experiences as “affectionless control” or “weak or absent” bonding (95), which was further associated with suicidal behavior (96). The most commonly reported adversities were emotional abuse, physical abuse, and witnessing household members being subjected violence (42.3%, 39.9%, and 34.6%, respectively), which showed dose-response relationships with poor mental health, including but not limited to, severe depression, anxiety, and reduced well-being and happiness (97).

##### 3.1.5.4 Low parent education or early parenthood

Lower education or early parenthood might lead to a greater likelihood of inadequate parenting, such as maltreatment or being rigid and restrictive (98), which result in poorer mental health among offspring. Maternal education was negatively associated with major depressive episodes in early-adulthood (99). Low parental education significantly predicted the persistence and severity of mental disorder (41). Indeed, having the lowest level of parental education continued to be the strongest risk factor for parent-reported child mental health problems (100). In addition, early parenthood was also confirmed as a predictor of later developmental disorder (101).

In discussion of parenting styles, parental indifference, parental overprotection, family abuse, and low parental education or early parenthood were regarded as risk factors.

#### 3.1.6 Parent-child relationship

As anticipated, the parent-child relationship significantly affected the well-being of offspring (35, 50). In general, the mother-child relationship exerted a stronger effect on children’s behavioral problems (102). The interaction between parents and children might form a “chain-reaction”: parent-child interaction induces children’s feelings toward parents, themselves, and interpersonal relationships, which in turn influences their levels of mental health.

##### 3.1.6.1 Insecure attachment

Attachment is shaped during infancy, which has far-reaching effects on offspring’s lifelong well-being. Insecure attachment is associated with problems including depression (103) and suicidal behavior (45). Specifically, “disorganized” patterns of attachment, have the strongest negative impact on both concurrent and later psychopathology in offspring (104).

##### 3.1.6.2 Alienation towards parents

Inadequate parenting and separation from parents might cause children’s feelings of alienation toward their parents. Researches have evaluated the link between emotional alienation toward parents and depression in left-behind children (LBC) using longitudinal designs (197) and have also highlighted the higher level of maternal alienation in LBC (105).

##### 3.1.6.3 Fear of family members

Individuals with frequent fear of a family member showed the strongest association between childhood adversities and the use of psychotropic drugs, and this association was stronger among those with poor parent-child relationships (106). Persistent mental disorders were found in 16.9% of pregnant women which were partly predicted by fear of a family member (T. D. 107).

##### 3.1.6.4 Separation with parents

Early maternal separation and isolation rearing has been linked to depressive behaviors (108). Individuals who experienced prolonged separation (i.e., lasting one year or longer) developed depressive systems in response to lower levels of life stress (109). Among adoptees, extended early deprivation was associated with long-term deleterious effects on well-being that appear resistant to years of nurturance and support in adoptive families (110). Importantly, parental death is a special and severe form of separation, which is a strong predictor of clinical depression in adolescents (92). Moreover, early-life parental death was associated with increased adult suicide risk beforeage50 (111).

### 3.2 Protective family factors for mental health of offspring

#### 3.2.1 Biological genetics

Animal study showed that variation in 5-HTT function was associated with vulnerability or resilience to adversity across the lifespan (112). Specifically, 5-HT1A and 5-HT1B receptors were both found to facilitate psychological resilience and antidepressant effects in depressed patients with childhood trauma (113). Self-perceived psychological resilience was associated with marked buffering of the activation of the conserved transcriptional response to adversity (CTRA, antiviral genes in circulating immune cells), such that PTSD-affected child soldiers with high levels of personal resilience showed unique molecular profiles compared to those of PTSD-free civilians (114).The results suggested that the function of the serotonin (5-HT) transporter gene and antiviral genes (CTRA) were protective factors for mental health outcomes in offspring.

#### 3.2.2 Family conditions

##### 3.2.2.1 Safe and stable family environment

Policies protecting unauthorized immigrant mothers resulted in improved mental health among their children (115 Certainly, the safe and stable nurturing environment was associated with reduced biological markers of toxic stress, as well as improved clinical outcomes (116).

##### 3.2.2.2 Familism and family pride

One’s perception of family values may be reflected in familism and a sense of family pride, both of which may serve as protective factors for the mental well-being of the younger generation. Familism emphasizes close family relationships, interconnectedness, and prioritizing family over the self (117). A previous study has shown that higher levels of familism predicted lower loneliness and depression among family members (118). Similarly, the related concept of *family pride*, which signifies one’s positive sentiment toward the family, is also associated with positive outcomes. It was found that family pride was associated with higher self-esteem, greater family-responsive happiness among adolescents who grew up in a collectivist cultural background (119), and greater experiences of joy among emerging adults (120).

##### 3.2.2.3 Social support of family

Children demonstrated better outcomes when their families believed they were part of a community with shared values and mutual obligations (121). Consistently, parents’ perceived support strongly influenced child-rearing practices. A supportive family relationship during a child’s upbringing can buffer responses to toxic stress that result from extreme or prolonged activation of the body’s stress-response systems in a maladaptive developmental environment (122, 123).

In conclusion, the safe and stable family environment, familism and familial pride, and family social support were protective factors.

#### 3.2.3 Parental mental wellness

Parental personality, as a stable behavioral pattern, plays a critical role in children’s development. Maturity parental traits such as calmness and cooperation helped children become well-adjusted (124), serving as a protective factor.

#### 3.2.4 Relationship between father and mother

##### 3.2.4.1 High levels of marital quality

Importantly, parents who were satisfied with their marriages served as more attractive role models for their children, making them more likely to emulate family-related ideals modeled by their happily married parents (125).

#### 3.2.5 Parenting style

As for types of parenting, maternal sensitivity softened the negative impact of early-adversity on children’s development (17).

##### 3.2.5.1 Coparenting

Coparenting has been identified as a key factor that promotes children’s psychological adjustment (126, 127). A child’s positive affect was significantly associated with maternal-perceived coparenting support (128). Moreover, coparenting quality positively affects parent’s marital relationship, which in turn benefits children’s mental outcomes. Specifically, parent’s coparenting quality was linked with both their own and their partner’s perceived relationship quality, including relationship adjustment and negative interactions (129).

##### 3.2.5.2 Parental warmth

Importantly, based on the 76-year longitudinal grant study, the warmth of childhood relationships with parents had the greatest positive impact on “life satisfaction” (130). High parental warmth was associated with a decreased risk of developing an insecure attachment style (103), as well as reduced anxiety and depression (131). Moreover, warm parenting was associated with a marked reduction in children’s internalizing and externalizing problems over time (132).

In brief, parental sensitivity, optimal coparenting and parental warmth were protective factors of offspring’s mental health.

#### 3.2.6 Parent-child relationship

##### 3.2.6.1 Secure attachment

Specifically, the touch-sensitive attachment between mother and infant contributed to the child’s long-term emotional and mental stability (133); maternal attachment softened the impact of early adversity (17). Importantly, a secure attachment to the father was not enough to reduce infant stress reactivity when the infant-mother attachment was insecure (134), indicates a mother preference again. However, the paternal role is still important. For example, Waldegrave (135) suggested that maintaining paternal involvement during imprisonment was vital in promoting and maintaining positive infant mental health, which might increase the infant’s attachment to the father. For adolescents, a secure attachment in infancy was associated with more appropriate emotional expression than that of insecure peer (136).

Regardless of whether or not they lived with two biological parents during childhood, individuals who perceived love from caregivers had significantly lower odds (by 42-43%) of lifetime suicidal thought (137).

Frequent contact with nonresidential parents helps reduce the risk of mental health symptoms (138). Moreover, higher-quality and more frequent interactions with biological parents predicted a delayed onset of sexual behaviors, which were linked to negative outcomes for adolescents (139).

Taken together, the results suggested that insecure attachment, alienation from parents, fear of family members, and separation from parents were risk factors, whereas secure attachment, frequent contact with parents, and perceived love from caregivers served as protective factors.

## 4 Potential mechanisms of family dominant hypothesis

Systematic evidence for the *family domain hypothesis* has been discussed above, for which different mechanisms might underlie the effects. Why did the family of origin influence children so profoundly? Neurobiological alteration might be an important mechanism. Genetic factors provide different starting points for infants; the interaction between genes and the environment promotes development across the lifespan. However, brain mechanisms have not been sufficiently discussed in combination with the family of origin.

### 4.1 Neural mechanisms

#### 4.1.1 Cerebral alteration

First of all, the immature brain might change owing to early raising experiences (140). For example, in the obsessive-compulsive disorder group, childhood trauma was positively associated with a larger right orbito-frontal cortex volume (141). The prefrontal cortex has extensive connections to subcortical areas, which might also explain the impact of the family of origin. For example, threat appraisal via the amygdala was maladaptive after early adversity because of the dysregulation of the amygdala and ventromedial PFC (P. 142). In addition, early adversity was associated with structural atrophy in the PFC and hippocampus, but hypertrophy of the amygdale (143).

Alternatively, a healthy family of origin might have a positive impact on the neurological development of offspring. For example, higher maternal warmth predicted lower neural activation in response criticism in the left amygdala, bilateral insula, and other related regions (144). In addition, positive maternal behaviors in childhood were associated with larger volumes in the frontal orbital gyrus (145) and the dorsolateral PFC in adults (146). Moreover, high levels of positive parenting style or quality were associated with reduced amygdala volumes (147, 148) and larger hippocampal volumes (149–152).

#### 4.1.2 Immunological response

Family of origin is associated with the immune system of an individual. For example, maternal distress was associated with the child’s neuroendocrine-immune markers in saliva, which might alter the sensitivity of inflammatory immune processes to the inhibitory effects of cortisol (153). Research generally agrees that immunity can affect an individual’s mental health (154, 155). Immune response might be a mediator between the family of origin and offspring’s mental well-being. For example, prolonged exposure to early adversity might lead to a more pro-inflammatory phenotype, which activates immune cells to release more inflammatory cytokines exacerbates the inflammatory response, with long-term adverse consequences for a range of mental health problems (156).

#### 4.1.3 Cortisol response

Specific early experiences are associated with characteristic early changes in the functionality of the glucocorticoid system and result in a predisposition to distinct mental and behavioral disorders (157). For example, in adoptees with an anxiety disorder, severe maltreatment was associated with lower daily cortisol secretion than that of non-maltreated adoptees (10).

However, alterations of hypothalamic-pituitary-adrenal axis activity and glucocorticoid receptor expression levels in the hippocampus did not occur significantly when experiencing adversity during both early-life and adulthood, which supported the notion that being raised in a stressful environment prepared the offspring to better cope with a challenging adult environment and emphasize the role of early-life experiences in shaping adult responsiveness to stress (158). In addition, Champagne et al. (159) found that early-life conditions prepared individuals for life ahead through glucocorticoid programming and phenotypic plasticity, with the goal of ‘match’ future environmental demands, suggesting that the glucocorticoid system may facilitate stress-related adaptation.

### 4.2 Potential causal interaction mechanism within family of origin factors

According to the Family System Theory, a family is a system composed of interdependent parts. Any change in one component (such as the parent-child or the parental relationship) will affect all other parts of the system (11). On the basis, family factors do not operate in isolation but demonstrate the potential for causal interactions. However, these relationships remain indecisive. *First*, distal family factors transmit influences to proximal ones, ultimately shaping offspring’s mental health. For example, poor family economic status undermines parent’s mental health and parenting style, which in turn contribute to offspring’s mental distress (13). Offspring’s well-being was associated with genetic factors, and that early environmental factors related to infant and toddler health moderated this association (160). Moreover, the family’s physical environment and parenting style, serving as mediating factors, were influenced by low family income and affected children’s intellectual development (161). Better parental mental health, especially maternal, was shown to weaken the negative association between family economic hardship and child mental health (162). Parental mental wellness mediated the relationship of family economic hardship (163)/residential instability (164) and adolescents’ internalizing and externalizing problems.

Second, family factors within the family system may interact with one another, jointly influencing offspring’s mental health. Specifically, the effect size of one family factor on offspring’s mental health would be amplified, attenuated, or operate in qualitatively different ways when interacted with another factor. For instance, parent’s rejection amplified the relationship between parent’s mental distress and offspring’s mental health (165). Similarly, parental behavioral control functioned in a context-dependent manner, enhancing the protective effect of a good parent- child relationship on adolescent mental health while exacerbating its adverse impact under conditions of poor parent-child relationship quality (166). Marital status influenced the parenting style (167), and parenting style mediated the association between parental and children’s mental health. Parent-child relationship quality buffered the impact of negative parenting style on child’s internalizing and externalizing symptoms (168). In particular, the relationship between parental depressive symptoms and child psychosocial problems was mediated by parenting behaviors (E. 169). Moreover, the link between parental divorce and children’s mental health was fully mediated by attachment style and childhood trauma (170). Parenting stress was related to more aggression and attention problem behaviors in insecurely attached children, but not in securely attached children (171). Parents’ marital satisfaction was associated with fathers’ higher levels of supportive coparenting behaviors, which in turn was associated with a decrease in child internalizing problems (172). Collectively, it is not difficult to discern that latent causal interactions among family variables likely exist, although these dynamics are not yet fully delineated. See **Supplementary Materials (Figure S1)**. Longitudinal investigations with path analysis are necessary to conduct in the future.

In brief, the influence of childhood experiences on offspring’s health-related outcomes may be closely intertwined with psychological and neurobiological mechanisms, suggesting potential interventions in clinic and psychological counseling settings.

## 5 Implications for preventive-therapeutic family interventions

Despite the effects of the family of origin on offspring, early recognition, prevention, and supportive measures may protect offspring from the negative impacts of early-family adversities (140). Since the family is the smallest functional unit of society, family-based intervention is particularly critical. Guided by the Family Dominant Hypothesis, interventions targeting at a single family factor may exert broader effects on offspring mental health by interrupting cascading risk pathways (173) or by modifying key mediating and moderating processes within the family system (82).

### 5.1 Interventions targeted at genetics

Genetic vulnerabilities in offspring do not only increase individual health risks (174) but may also impose substantial emotional and financial burdens on parents, thereby altering family climate, parenting quality, and overall family functioning (175). In this sense, prenatal genetic screening serves not merely as a biomedical preventive strategy, but as a starting point of family-level intervention. To identify individuals at high genetic risk prior to pregnancy, Rowley (176) urged public and professional education on human genetics. Indeed, prenatal carrier screening and diagnosis using DNA-based molecular methods have become crucial in early detection and intervention (177). Guided by the Family Dominant Hypothesis, prenatal gene screening would allow couples to make better preparations and choices regarding whether to have a child (178), stabilize family functioning and reduce cascading risks to offspring mental health.

### 5.2 Interventions targeted at family conditions

According to the Family Dominant Hypothesis, family conditions mainly consist of two aspects: family economic conditions and negative life events, which are difficult to modify directly through psychological interventions. Importantly, family conditions such as economic hardship or adverse life-events have indirect impacts on offspring mental health (163, 164), which align with Ecological Systems Theory (19). As such, the negative impact of family conditions can be mitigated or even intercepted by intervening in proximal family process. For example, a harmonious family encompasses aspects such as the mental health of parents, good parent-child relationships and parent-child interactions, and appropriate parenting styles, may buffer the effects of adverse conditions (179, 180). Therefore, both psychological interventions and family-oriented policies should prioritize strengthening modifiable intra-family processes to intercept the impact of family conditions (173).

### 5.3 Interventions targeted at parental mental wellness

Previous studies revealed that parental mental wellness represents a mediating pathway through which distal family stressors are translated into parenting practices (163, 164). Therefore, improving parental mental wellness may not only reduce transmission of distal family stressors but enhance parents’ capacity to buffer stress, thereby modifying the associations between other proximal family factors and children’s mental health. Providing psychological counseling to expectant parents during pregnancy is important for promoting the health of new families. A previous review suggested that parental depression negatively impacted offspring development from the antenatal period to adulthood, which could be effectively prevented by parental mental health screening during pregnancy (181). Moreover, it has been strongly recommended that all pregnant women identified as being at high risk should have a shared care plan for psychiatric management during late pregnancy and the early postnatal period (182). However, for parents who experience mental health issues or substance use problems after childbirth, the intervention focuses mainly on their children (183). Importantly, parents of children with developmental delays who participated in mindfulness-based stress reduction (MBSR) reported significantly less stress and depression, along with fewer behavioral problems in their children following the intervention (184). The results confirmed the effectiveness of the intervention for parental mental disorders and highlighted the need for more practical implementation, which remains limited at present. Finally, for families with a history of mental illness, it is important to seek professional psychological counseling at the early stages of family formation and when preparing to raise a child.

### 5.4 Interventions targeted at relationship between father and mother

Currently, marriage therapy is commonly practiced. Evidence shows that marriage therapy improves marital satisfaction, reduces marital conflict over child rearing, and enhances child adjustment (185). Importantly, according to Family Dominant Hypothesis, the relationship between parents encompasses not only the marital relationship but also the cooperative relationship in raising children (186). Therefore, marriage therapy should be conceptualized not merely as a couple-focused intervention but as a family-level strategy. Evidence showed that compared with behavioral couples therapy alone, behavioral couples therapy combined with parent skills training resulted in a larger effect size on parenting and involvement with child protection services (187). These findings support the use of marriage therapy for parents experiencing marital discord to promote a more positive family environment for their children. By reducing parental conflict and enhancing parental cooperation, they may prevent relational stress from spilling over into parenting practices and the parent-child relationship, thereby mitigating its negative impact on offspring’s mental health (172). Accordingly, interventions targeting the parental relationship may yield amplified effects by simultaneously improving multiple family subsystems, rather than operating solely at the couple level.

### 5.5 Interventions targeted at parent-child relationship and parenting style

As integral participants in a child’s treatment, parents may play a more effective role in the treatment of young children who are exposed to multiple risks (188). According to the Family Dominant Hypothesis, the parent-child relationship constitutes a primary pathway through which family processes influence offspring mental health, suggesting that interventions should prioritize strengthening the parent-child interaction quality. Child-Parent Psychotherapy (CPP) (189, 190) is an empirically supported treatment in which the child and primary caregiver participate jointly, with the therapeutic goal of enhancing the parent’s capacity to provide safety and developmentally appropriate care giving to the child. Originating in Fraiberg’s infant–parent psychotherapy (191) and attachment theory (30), CPP has been extended to the treatment of children exposed to violence and other traumatic stressors (188). Results confirmed the effectiveness of family-centered care in improving parent-child relationship and parenting styles (69). By enhancing parent-child interactions and modifying maladaptive parenting practices, CPP may help shape offspring’s internal working models (192), thereby promoting more adaptive mental health outcomes. Moreover, improvements in parenting practices and parent-child relationship quality may modify how other family risk factors affect children, functioning as a protective moderator within the family system (172).

## 6 Open questions: *gender difference*

Regarding the potential gender differences, some findings have supported the female vulnerability hypothesis. For example, the analysis showed that being female emerged the strongest predictor of mood and anxiety disorders after experiencing early family adversity (6). Independently, some findings have supported the male vulnerability hypothesis. For example, boys who experienced adverse childhood events were more likely to engage in antisocial behavior in young adulthood (193). Boys seemed prone to depression after their parents’ divorce, regardless of the quality of parenting they received (194). Alternatively, the no-gender-difference hypothesis was also suggested; for example, maternal early-life stress contributed to altered cortisol responses at a similar rate in males and females (54). Thus, we assumed that gender differences in the effect of the family of origin on offspring might not exist. The observed gender effect in some studies might be due to the following reasons. (1) Females tended to report more mild psychological problems even when they experienced similar symptoms compared with males (195). (2) Alternatively, the gender effect of early-life adversity on offspring might be caused by the specific influence of parental gender. For example, boys were more affected by parental divorce, probably because most of them lived with their mothers after their parents’ divorce, which deprived them of the chance to interact closely with their fathers, who shared the same gender as the boys (194). (3) Alternatively, the observed gender effect might also be due to variations in symptom phenotypes between genders, *i.e.*, different symptoms of the same disorder in the two genders (196). More studies are needed to confirm this conjecture.

In conclusion, we propose a family dominant theory to address the importance of a healthy family of origin in protecting offspring’s well-being. In this Family Dominant Hypothesis, family of origin and environmental variables, encompassing both risk and protective factors, contribute to different mental health outcomes in offspring. Specifically, the family of origin plays both a direct and mediating role in children’s development. The significance of this theoretical framework is to emphasize the leading and mediating role of the family of origin in both risk and protective contexts. The aim of this review is to call on society to pay attention to the dominant and mediating role of the family of origin, which requires immediate action from families and young parents (e.g., discontinuation of inappropriate parenting and timely corrective actions) to ensure better mental health outcomes of the next generation.

## Data Availability

The original contributions presented in the study are included in the article/**Supplementary Material**. Further inquiries can be directed to the corresponding author.

## Supplementary material

Supplementary Table 1 Evidence of Risk and protective family factors for mental health of offspring.

Supplementary Figure 1 Latent causal interactions among family variables based on Family Dominant Hypothesis.
