# Supplementary material for: Family dominant hypothesis for the effect of family of origin on the mental health of offspring: evidence, mechanism, and implications
Source: Front Psychiatry. 2026 Jan 28;17:1733077. doi: 10.3389/fpsyt.2026.1733077 (PMC12891180; doi:10.3389/fpsyt.2026.1733077)
Supplement: Supplementary Table 1 — Evidence of Risk and protective family factors for mental health of offspring. [file Table1.docx]

Supplementary material

| Table S1. Evidence of Risk and protective family factors for mental health of offspring. | | | | | | | |
| --- | --- | --- | --- | --- | --- | --- | --- |
| **Studies** | **Measured variable related to family factors** | **Measured mental health outcome** | **Sample** | **Statistics analysis** | **Finding’s supportive effect of family on offspring** | | **Effect size** |
| **Biological genetics** | | | | | | | |
| Bleys et al. (2018) | **1.**5-HTTLPR  **2.Stress** | **Depression** | N = 51 studies  (Meet the standards) | Meta analysis | ***Limitedyes:** 5-HTTLPR x stress had small but significant interaction effects on depression  Risk factor: Risk factor x Stress | | Overall: OR(95%CI) = 1.18(1.09; 1.28) |
| Lipsky et al.(2022) | 1.Serotonergic signaling system  2.Adverse childhood experiences (ACEs) | Depressive and anxiety disorders | N = 28 studies  (Meet the standards) | Meta analysis | ***Yes:**5-HTTLPR moderated the association between ACEs and depression | | N/A |
| Simonyte et al. (2023) | 1.Stressful life events  (List of Threatening Events Questionnaire)  2.5-HTTLPR | Depression  (EURO-D scale) | N = 353, aged 65 years and over | Multivariate logistic regression | ***Yes:**5-HTTLPR moderated the association between stress events and depression.  Risk factor: s-allele carriers exposed to stress | | OR (95% CI) = 6.899 (2.903–16.393), *p*< 0.001 |
| Byrd andManuck (2014) | **1. MAOA genotype**  **2. Early-life adversity** | **Aggressive and antisocial behavior** | N = 27 studies  (Meet the standards)  Only male participants (12); mixed participants(11); only female participants(4). | Meta analysis based on Liptak-Stouffer z score procedure | ***Broadly yes:** Significant early adversity x MAOA genotype (G x E) interaction for male cohorts.  Risk factor: MAOA | | *p* = 0.0044 |
| Zhang et al. (2016) | 1. MAOA genotype  (DNA collection)  2. Childhood maltreatment  (Childhood Trauma Questionnaire-Short Form, CTQ-SF) | Aggression  (Youth Self-report questionnaire, a self-reporting version of the Child Behavior Checklist) | N = 507 male, mean age =15.81 years. | t tests, Linear regression | ***Limitedyes:** A significant childhood maltreatment x MAOA genotypes (G x E) interaction, while the main effect of MAOA was not significant.  Risk factor: MAOA | | Abuse and Abuse x MAOA: Adj R²=22.5%, *p* = 0.008 |
| Xu et al. (2022) | 1.Genotype  (DNA Methylation)  2.HTR1A and HTR1B receptors  3. Stress  (Life Events Scale, LES; Childhood Trauma Questionnaire, CTQ) | **Antidepressant efficacy** | N=391, 18-65 years old. | t-test, Mann–Whitney U-test, chi-square test, Logistic regression | ***Yes:**1.Low HTR1A-2-143 methylation combined with low CTQ score was related  to better antidepressant efficacy; 2. HTR1B methylation with the rs6298 AA/AG genotype affects better antidepressant efficacy.  Protective factor: 5- HTT | | 1.OR (95% CI) = 0.459(0.268 , 0.785), *p* = 0.004.  2.OR (95% CI) = 2.030(1.222 , 3.372), *p* = 0.006. |
| Kohrt et al. (2016) | **CTRA Gene Expression** | 1.PTSD  (Child PTSD Symptom Scale)  2.Resilience  (Wagnild and Young’s Resilience Scale) | N = 290, 15-26 years old.  Groups (n): Former child soldiers(154); civilian children(136). | ttests, Chi-square tests, mixed-effect linear model analyses. | ***Yes:** CTRA responses to early-life adversity underscore the key role of resilience in determining the molecular impact of adverse environments.  Protective factor: CTRA | | N/A |
| **Family conditions** | |  |  |  |  | |  |
| Hyland et al. (2016) | Deprivation  (>21 days unemployment) | Mood and anxiety disorder ( ICD-10 diagnosis) | N = 54,458 new born and their parents.  Groups(n): anxiety disorder(1,772), mood disorder(790). Tested in the period of 1994-2005.  Longitudinal samples (1984-2005). | Chi-square tests, multivariate logistic regression. | ***Yes:** Parental unemployment predicted offspring's mood and anxiety disorder.  Risk factor: Family poverty | | 1.Anxiety disorder: OR(95% IC)=1.26 (1.13–1.41)  2.Mood disorder: OR(95% IC) = 1.19 (1.01–1.41) |
| McLaughlin et al. (2011) | Child SES  (Parental education; Financial hardship; Parental occupation) | Onset, persistence and severity of mental disorders  (NCS-R diagnoses) | N = 5,692, 18 years old. | logistic regression. | ***Yes:**1. Parental occupation predicted disorder s onset in childhood; 2. financial hardship predicted disorders onset at every life-course stage; 3. financial hardship predicted disorders severity.  Risk factor: Family poverty | | 1.χ_3_^2^ = 8.9, *p* = 0.03.  2.OR = 1.4-2.3, p<0.05.  3.OR = 1.7, p<0.05. |
| Rahman et al. (2009) | Experience of childhood poverty  (Semi-structured interview) | Psychological distress  (Self-Reporting Questionnaire, SRQ-20) | N = 337 females, 16-18 years old. | Multiple regression. | ***Yes:** Experience childhood povertypredicted the psychological distress for female adolescents.  Risk factor: Family poverty | | N/A  Beta = 0.122, p = 0.028 |
| Wagner et al. (2009) | Socioeconomic Status  (Mother education level and family income) | 1. Behavior problem symptom 2. (Health and Behavior Questionnaire) 3. 2. Disruptivebehaviors   (DISC-IV) | N=750, 7-8 year old twins | Hierarchical Linear Modeling. | ***Yes:** Family income significantly predicted child's conduct and ADHD symptoms.  Negative: Maternal education level did not significantly predicted child’s conduct and ADHD symptoms.  Risk factor: Family poverty | | N/A  1. Family income → conduct:  Beta = -0.082, p<0.01  2. Family income → ADHD symptoms:  Beta = -0.037, p = 0.02 |
| Gilman et al. (2003) | 1. Childhood SES   (Occupation)  2. Residential instability  (Number of moves) | 1. Age at onset of major depressive episode; 2. Lifetime depression, recurrence or remission. 3. (Diagnostic Interview Schedule, DIS) | N = 1,089, 18-39 years old. | Poisson regression. | ***Yes:** Childhood SES significantly influences risk of depression onset both in childhood and in adulthood, residential instability significantly influences risk of depression onset in childhood (≤ 14 years old).  Risk factor: Family poverty | | 1. Occupation:   HR(95% IC) = 1.57(1.08,2.29)   1. Residential instability:   HR(95% IC) =2.62(1.56, 4.39) |
| Hainmueller et al. (2017) | Unauthorized immigrants | Mental health  (International Classification of Diseases 9, ICD-9) | Used data from  Emergency Medicaid | Local linear regression. | ***Yes:** Mothers’ DACA eligibility significantly decreased adjustment and anxiety disorder diagnoses among their children.  Protective factor: Safe and stable family environment | | N/A |
| Najman et al. (2017) | Adverse life events for mothers  ( 9 questions from Holmes and Rahe, 1967) | Depression  (depression subscale of Delusions-Symptoms-States Inventory, DSSI) | Mother: N=6,753 → 3,561  Children:N = 7,223 → 2,900  Longitudinal samples | [Generalised estimating equation, Poisson regression, multinominal logistic regression model](https://www.sciencedirect.com/topics/medicine-and-dentistry/logistic-regression-analysis). | ***Yes:** Children reared in a family experiencing high levels of adverse life events were more likely to experience a lifetime depression.  Risk factor: Adverse family event | | Adj OR = 1.46-1.97 |
| Wall-Wieler et al. (2016) | Health conditions (0-13 years old)  (ADHD, conduct disorder/ODD, bipolar disorder, asthma, major injuries, others) | **ADHD**(14-18 years old) | N = 62,739.  sibling (N = 29,444) | Multilevel model. | ***Mostly yes:** Health conditions had significantly effects on late adolescent ADHD in original cohorts mostly (excepted major unjuries) and on sibling in varying degree.  Risk factor: Poor health conditions | | 1. Original cohorts: OR = 1.18 - 25.23.  2. Sibling: OR= 1.22 - 16.01. |
| Corona et al. (2017) | **Familism**  (14-item Familism scale) | 1. Loneliness 2. (20-item Loneliness Scale) 3. 2. Depression   (CES-D) | N =811. | Regression; ANOVA and Bonferroni post hoc tests. | ***Yes:** Familism was negatively associated with loneliness and depression.  Protective factor: Familism | | N/A  1.For loneliness  B = -0.14, *p*<0.01  2.For depression  B = -0.50, *p*<0.05 |
| Patel et al. (2022) | **Familial cultural values**  (Recently articulated construct of familial pride) | 1. **Depression** 2. **2. Self-esteem** 3. **3. Family-responsive joy** | N = 219, 18-28 years old. | Path analyses. | ***Yes:** Familial pride was uniquely associated with lower depressive symptoms, higher self-esteem, and higher family responsive joy.  Protective factor: Familial pride | | 1. Familial pride→ depression   B = -0.101, *p*<0.001   1. Familial pride→ self-esteem   B = 0.078, *p*<0.01   1. Familial pride→ family responsive joy   B = 0.245, *p*<0.001 |
| Stein et al. (2019) | 1. **Familism pride**   **(**Self- made familism pride measure)   1. **Familial cultural values**   (Mexican American Cultural Values Scale, MACVS) | Depressive symptoms  (10-itemversion of Center forEpidemiological Studies Depression Scale, CES-D; Eight-item version of the Patient Health Questionnaire (PHQ-9) | N = 667,18-28 years old. | Path analyses. | ***Yes:**Familial pride predicted lower depression symptom.  Protective factor: Familial pride | | B = - 0.24, *p*<0.01 |
| Runyan et al. (1998) | Social capital (Five indicators) | Child well-being  (Battelle Developmental Inventory Screening Test,BDST) | N = 667, 2-5years old children and their maternal caregivers | Logistic regression. | ***Yes:** Social capital has a protective impact on children's well-being.  Protective factor: Social support of family | | The odds increases for doing well: 29% by one social capital indicator, 66% by any two social capital indicator. |
| Shonkoffand Garner (2012) | Social support (Family APGAR) | Psychosocial dysfunction  (Pediatric Symptom Checklist, PSC and physician assess) | N = parents of 9626 children (4 -15 years old) | Chi-square tests，ANOVA. | ***Yes:** lack of social support was more likely to trigger psychosocial dysfunctions of children whatever through PSC or physician assessment.  Protective factor: Social support of family | | 1. PSC assessment: OR = 2.2 2. 2. Physician assessment: OR = 4.3 |
| **Parental mental wellness** | |  |  |  | |  |  |
| Hyland et al. (2016) | Paternal and maternal history of any mood or anxiety disorder  (ICD-10 diagnosis) | Anxiety and mood disorders in children  (ICD-10) | N = 58,489, retrospective, longitudinal samples. | Binary logistic regression analysis. | | ***Yes:** A parental history of a mood or anxiety disorder was the strongest predictor of mood and anxiety disorders.  Risk factor: Parental psychiatric disorder | 1. Mood disorder: OR = 1.65-2.04 2. Anxiety disorder: OR = 1.51 - 2.38 |
| Hayden and Klein (2001) | Family History  (Family History Research Diagnostic Criteria interview;Family History Interview for Personality Disorders; Direct interviews with relatives using the SCID; The Personality Disorder Examination) | Depression  (Hamilton depression scale) | N = 86 outpatients with a DSM-III-R diagnosis of dysthymic disorder, primary and early-onset types. Longitudinal samples. | Hierarchical multiple regression. | | ***Yes:** Family history of dysthymic disorder predicted higher levels of depression at follow-up.  Risk factor: Parental psychiatric disorder | R² = 0.13, F = 10.45, *p<*0.01 |
| Robinson et al. (2022) | **Parental depression** | **ADHD diagnosis and symptom outcomes of offspring** | N = 58 studies  (Meet the standards) | Meta analysis | | ***Broadly Yes:** Parental depression was significantly associated with ADHD symptoms.  Risk factor: Parental psychiatric disorder (Depression) | 1. For inattentive symptoms:  CC(95% CI) = 0.10 (0.05, 0.15)  2. Forhyperactive/impulsive symptoms:  CC(95% CI) = 0.12(0.05, 0.19) |
| Rahman et al. (2009) | Current major depressive episode of mother  (Structured Clinical Interview for DSM-IV Disorders, SCID) | 1.Current Major Depressive Episode of female adolescents (Structured Clinical Interview for DSM-IV Disorders, SCID)  2.Psychological distress(SRQ-20) | N = 337 females(16-18 years old) and all mothers. | Correlates, Multiple regression | | ***Weakly yes:**Maternal depression predicted offspring's psychological distress.  Risk factor: Maternal depression | N/A  Beta = 0.105, *p* = 0.048 |
| Collin et al. (2015) | Maternal anxiety and depression  (Edinburgh Postnatal Depression Scale, EPDS; Crown-Crisp Experiential Index, CCEI) | Chronic disabling fatigue  (reported by parents through questions) | N = 14,541pregnancies and 13,978 children.  longitudinal samples. | Chi-square tests, *t* tests, logistic regression. | | ***Yes:** Children whose mothers experience anxiety and/or depression have an increased risk of developing chronic disabling fatigue in early adolescence.  Risk factor: Maternal anxiety and depression | 1. Maternal anxiety: OR = 1.19  2. Maternal depression: OR = 1.24 |
| Epperson et al. (2017) | Maternal adversity  (Reported experiencing at least two adverse childhood experiences before the age of 18 were considered High ACE Group) | Child cortisol  (Salivary cortisol) | N = 90 | Mixed-model ANOVA. | | ***Yes:** With High ACE women, on average, experiencing a 50% lower cortisol response to the infant separation paradigm than did low ACE women. Infants of High ACE mothers also demonstrated lower cortisol response to restraint and noise stress.  Risk factor: Maternal early-life stress | N/A |
| Madigan et al. (2018) | Prenatal stress  (depression and anxiety) | **Socio-emotional problems** | N = 73 studies  (Meet the standards) | Meta analysis | | ***Broadly yes:** Maternal prenatal stress was associated with offspring socio-emotional development  Risk factor: Maternal prenatal stress(anxiety and depression) | 1. Depression:   OR (95% CI ) = 1.79 (1.61-1.99)   1. Anxiety:   OR (95% CI ) = 1.50 (1.36-1.64) |
| Murray et al. (2011) | Maternal Mental State  (Standardized Psychiatric Interview, SPI at recruitment; Schedule for Affective Disorder andSchizophrenia—Life-time version at 18 months and 5 years; Structured  Clinical Interview for DSM-IV at 8, 13, and 16 years) | Offspring Mental State  (Kiddie Schedule for Affective  Disorders and Schizophrenia, Present and Lifetime Version, KSADs) | N(mother and their children) = 100 → 93, longitudinal samples. | Logistic regression, ANOVA, path analyses | | ***Yes:** Children of postnatal depressed mothers were more likely than controls to experiencedepression by 16 years.  Risk factor: Maternal postnatal stress (depression) | OR (95% IC) = 4.99 (1.68-14.70) |
| Evans et al. (2009) | Maternal depression  (Edinburgh Postnatal Depression Scale, EPDS) | Emotionality  (emotionality subscale of the Strengths and Difficulties Questionnaire, SDQ) | N = mothers of 8,431 children, aged 6–7 years. | Linear regression | | ***Yes:**Maternal depression increased the odds of high emotionality in the children  Risk factor: Maternal depression | OR (95% CI) = 1.99 (1.73-2.29) |
| Kvalevaag et al. (2013) | Fathers’ mental health  (Hopkins Symptom Checklist) | 1. Mental health in children 2. (The Strengths and Difficulties Questionnaire) 3. 2. Socio-emotional problems 4. (Infant Toddler Social and Emotional Assessment) 5. 3. Mainly behavioral and emotional developmental difficulties in children   (Child Behavioral Checklist Revised) | Children = 31,663, fathers = 31,458, data from the Norwegian Mother and Child Cohort Study. | Linear multiple regression and logistic regression models. | | ***Weakly yes:** Children whose fathers had high levels of psychological distress have higher levels of emotional and behavioral problems.  Risk factor: Paternal psychological distress | N/A   1. Fathers' psychological distress → Children's behavioral difficulties:   B(95% CI) = 0.19(0.15-0.23)   1. Emotional difficulties:   B(95% CI) = 0.22(0.18-0.26)   1. Social functioning:   B(95% CI) = 0.12(0.07,0.16) |
| Sifaki et al. (2021) | Paternal psychological distress  (Kessler Psychological Distress scale, K-6) | Child behavioral/emotional difficulties  (SDQ) | N = 19,243 UK families (19,517 children)  longitudinal samples. | Cross-lagged structural equation path models. | | ***Partly yes:**Paternal psychological distress predicted both subsequent child emotional and behavioral difficulties.  Risk factor: Paternal psychological distress | N/A  1.For child emotional difficulty：  At all ages:  B = 0.01 - 0.02, *p*_min_*<*0.05  2.For child conduct problems:  Age 5-7:  B= 0.01, *p <*0.05  Age 11-14: B = 0.02, *p <*0.001  3.For child hyperactivity/inattention:  Age 3-5: B = 0.02, *p <*0.01  Age 11-14: B = 0.01, *p <*0.01 |
| Kernreiter et al. (2020) | Parents’ mental health  German version of the Brief Symptom Inventory (BSI-18) | **Children’s mental health**  German versions of the Child Behavior Checklist. | N = 97 adoptive parents and their children (2-17 years old). | Univariate linear regressions | | ***Yes**: Emotional instability in adoptive parents affected a child's mental wellbeing.  Risk factor: Parental psychiatric disorder. | N/A  beta = 0.34, *p* = 0.002 |
| Sorocco et al. (2015) | Family history  (Family History Research Diagnostic Criteria, FH-RDC) | 1. Affect regulation 2. (Positive Affect and Negative Affect Scale, PANAS) 3. 2. Depression 4. (DIS-IV; BDI-II) | N = 599, 18-30 years old. | ANOVA | | ***Yes:** Family history of alcohol or substance use was associated with poor affect regulation and negative mood of offspring.  Risk factor: Family history of addiction | N/A  1. For affect regulation: F = 9.85, *p*< 0.001  2. For Depression (BDI): F = 22.38, *p<*0.0001 |
| RavajaandKeltikangas-Järvinen (2001) | Alcohol consumption and smoking  (self-reported by the parents) | Offspring temperament and character  (TCI) | N = 1,849 families, longitudinal samples. | General Linear  Model | | ***Partly yes:** Parental frequency of alcohol intake, getting drunk and smoking were associated with offspring temperament and character dimensions in varying degree.  Risk factor: Family history of addiction | N/A  1.Mother → Child  ①Frequency ofalcohol intake or getting dunking at year 3 → Child novelty seeking (F = 6.21, *p <*0.001, F = 13.14, *p <*0.001, respectively)  ②Frequency ofalcohol intake or getting dunking at year 3 → Child self-transcendence (F = 5.09, p *<*0.001, F = 5.46, *p* = 0 .004, respectively)  ③Frequency of smoking → Child novelty seeking (F = 6.16, *p* = 0.002)  2.Father → Child  ① Frequency ofalcohol intake or getting dunking at year 3 → Child novelty seeking (F = 5.72, *p <*0.001, F = 14.53, *p <*0.001, respectively)  ②Frequency ofalcohol intake→ Child self-transcendence (F = 3.56, *p* = 0.007) |
| Bolat et al. (2017) | Maternal and paternal personality  (Temperament and Character Inventory) | Adolescent psychopathology  (DSM-V) | The study group: 71 adolescents and 117 parents  The control group: 71 adolescents and 119 parents | χ^2^ test, Logistic regression analysis | | ***Yes:** Higher maternal HA (harm-avoidance) and lower paternal SD (self-directedness) were associated with adolescent suicide respectively.  Risk factor: High harm avoidance  Risk factor: Low self-directedness | 1. Maternal HA:   OR(95%CI) = 1.238 (1.046,1.465)  2. Paternal SD:  OR(95% CI) = 0.919 (0.850,0.993) |
| Kadak et al. (2015) | Self-directedness  (Temperament and Character Inventory, TCI) | Autistic traits  (The Autism Spectrum Quotient, AQ) | N = 70 parents of 40 children with autism spectrum disorder | Stepwise Regression | | ***Yes:** Low self-directedness predicted potential autistic trait for the offspring.  Risk factor: Low self-directedness. | N/A  B = - 0.251, *p* = 0.005 |
| Lee et al. (2015) | Parental personality profiles (Temperament and Character Inventory) | Children's behavior problems  (The Child Behavior Checklist 1.5-5) | N = 190, preschool outpatients and their parents | Correlation, stepwise regression analysis | | ***Yes:** The maturity of parents' character appears to have a key role in reducing the risk of behavior problems in their children.  Protective factor: Mature characters | N/A   1. SD(mother) → child behavior problems:   B = -0.94  2.ST(father) → child behavior problems  B = -0.184 |
| **Relationship between father and mother** | |  |  |  | |  |  |
| Hyland et al. (2016) | Family dissolution  (Defined as the child having experienced divorce, separation, and/or the death of a parent in a particular year) | Mood and anxiety disorders  (ICD-10) | N = 54,458 | Chi-square tests, multivariate logistic regression | | ***Yes:** Family dissolution increased the likelihood of mood and anxiety disorders.  Risk factor: Family disruption | 1. For mood disorder: 2. OR(95%CI) = 1.53 (1.30–1.79), *p<*0.001 3. 2. For anxiety disorder: 4. OR(95%CI) = 1.64 (1.47–1.83), *p<*0.001 |
| Fergusson et al. (2000) | Parental changes  (A parental change was counted if a parent left or entered the family home as a result of parental separation,divorce, death, remarriage, or reconciliation) | **Suicidal ideation**  (Self-reported) | N = 1,265  longitudinal samples | Proportional hazards regression | | ***Weakly yes:** Number of parental change was positively linked with suicidal ideation of offspring (ages between 15-21)  Risk factor: Family disruption | N/A  B = 0.06, *p<*0.05 |
| Gilman et al. (2003) | Family disruption  (Defined as either parental divorce or separation between the child’s birth and seventh year) | Lifetime depression  (DIS) | N = 1,089, 18 - 39 years old. | Poisson regression | | ***Partly yes:** Family dissolution was related to elevated lifetime risks of depression.  Risk factor: Family disruption | Model 1:  HR(95%CI) = 1.41(1.09-1.84), *p<*0.01.  Model 2 (Effects varying by age at depression onset): Only onset age ≤14:  HR(95%CI) = 2.39 (1.46-3.91), *p<*0.001. |
| Whitton et al. (2008) | Parental divorce and conflict  (Self-Reported) | **Marital interaction patterns**  **Adult psychopathology** | N = 265 couples | Correlation, hierarchical Regression Model | | ***Partlyyes:**Parental divorce only predicted women’s relationship commitment and confidence rather than couple.  Negative: Parental conflict did not significantly influence relationship commitment and confidence for both men and women.  Risk factor: Parental divorce | N/A   1. Parental divorce → women’s relationship commitment:   B = -0.12, *p <*0.05   1. Parental divorce → women’s confidence:   B = -0.14, *p <*0.05 |
| Donahue et al. (2010) | **Parents' relationship instability**  (Self-Reported: Separation or divorce of biological parents by age 5 was coded as early relationship  instability.) | 1. **Adolescents' history of sexual**   **partnerships**  (Self-Reported)   1. **Major depression**   (Computerized Diagnostic Interview Schedule IV) | N = 585 | Multivariate logistic regression analyses | | ***Yes:** Experiencing parents' relationship instability in early childhood was associated with sexual behavior or major depression in adolescence.  Risk factor: Parental marriage instability | 1. For sexual partnerships at age 16. 2. Model 4: OR(95%CI) = 1.92(1.15,3.22) 3. 2. For an episode of major depression during adolescence. 4. Model 5: OR(95%CI) = 2.61(1.17,5.81) |
| O'Connor et al. (2000) | **Family Type** | Psychosocial Risks  (Child Temperament Inventory) | N = 10,431 families | Logistic regression | | ***Weakly yes:** Exposure to psychosocial risks were elevated in single-parent families and stepfamilies, compared with intact or non-stepfamilies.  Risk factor: Family disruption | N/A |
| Riggio (2004) | Parental Conflict  (Parental Conflict Scale, PCS) | Relational anxiety  (The Anxiety Subscale of the Relationship Awareness Scale, RAS-A) | N = 566 | Correlation | | ***Weakly yes:** Parental conflict was linked with offspring's relational anxiety.  Risk factor: Parental conflict | r = 0.09, *p <*0.05 |
| Rahman et al. (2009) | Family factors  (The Index of Family Relations, IFR) | Psychological distress  (SRQ-20) | N = 321 female | Multiple regression | | ***Yes:**High SRQ score was associated with disturbed family relationships.  Risk factor: Marital discord | N/A  Beta = 0.295, *p<*0.001 |
| Cunningham and Thornton (2006) | 1.Maternal marital quality  (5 questions)  2.Children’s reports of parents’ marital quality  (2 questions) | Marriage-Related Attitude Measures  (7 questions) | N = 775 mother-child pairs (679 children eligible) | Regression | | ***Partly Yes:**Children’s reports of parent’s marital quality negatively influenced their support for divorce and premarital sex,while maternal reported marital quality had no significant effect.  Protective factor: High marital quality | Children’s reports of parent’s marital quality→ Children’s Attitude of divorce and premarital sex:  B = -0.10, *p <*0.01 |
| **Parenting style** |  |  |  |  | |  |  |
| Brown and Harris (1993) | Parental indifference  (Detailed questions covered possiblephysical or emotional neglect before the age of 17) | 1. Psychiatric symptoms   (Present State Examination, PSE)   1. Generalized Anxiety Disorder   (DSM-III-R) | N = 404 women,longitudinal samples. | Difference test | | ***Yes:**Parental indifference was positively associated with depression and anxiety.  Risk factor: Parental indifference | N/A  1.For anxiety: *p <*0.001  2.For depression: *p <*0.01 |
| Hill et al. (2000) | Parental care  (Parental Bonding Instrument,PBI) | Current affective symptoms  (Depression scale of the GHQ-28) | N = 862 women | Logistic regression | | ***Partly yes:** Low maternal care predicted depression.  Risk factor: Parental indifference | OR(95%CI) = 2.57 (1.70,3.90) |
| Otani et al. (2012) | Parental over-protection  (PBI) | Sociotropy  (Sociotropy–Autonomy Scale) | N = 416 | Multiple regression | | ***Partly yes:**High maternal protection predicted a high level of sociotropy for only females.  Risk factor: Parental over-protection (Mother) | B = 0.308, *p <*0.01 |
| Ong et al. (2011) | Parental over-protection  (PBI) | 1. Psychiatric disorders   (DSM-IV)   1. Heart-focused anxiety   (Cardiac Anxiety Questionnaire, CAQ) | N = 190 | Multiple regression | | ***Yes:**Parental over-protection predicted heart-focused anxiety.  Risk factor: Parental over-protection | N/A  B = 0.09, *p* = 0.02 |
| Otani et al. (2013) | Parental over-protection  (PBI) | Dysfunctional attitudes  (DAS-24) | N = 665 | Multiple regression | | ***Partly yes:** High maternal protection predicted high level of dysfunctional attitudes in achievement and dependency for only females.  Risk factor: Parental over-protection (Mother) | N/A  1.Maternal protection →achievement  B = 0.293, *p <*0.01  2.Maternal protection →dependency  B = 0.224, *p <*0.05 |
| Janssens et al. (2009) | Parental over-protection  (EMBU-C) | Functional somatic symptoms  (Youth Self Report, YSR) | N = 2,230  longitudinal samples | Regression | | ***Partly yes:** Maternal over-protection predicted functional somatic symptoms for only females.  Risk factor: Parental over-protection (Mother) | R^2^ = 0.18  Beta = 0.072, *p <*0.05 |
| Shibata et al. (2016) | Parental over-protection  (PBI) | Sleep disturbance  (Pittsburgh Sleep Quality Index, PSQI) | N = 702 | Mann-Whitney U test | | ***Weakly yes:**Paternal over-protection predicted only male’s sleep disturbance.  Risk factor: Parental over-protection (Father) | OR (95 % CI) = 2.17 (1.03–4.58)  *p =* 0.04 |
| Lima et al. (2014) | Parental behavior and healthy parent-child bonds  (PBI) | **SCID, cortisol levels** | N = 133, 71 with PTSD, 62 without PTSD. | Chi-square tests, ANOVA, FBST – Full Bayesian Significance Test. | | ***Yes:** Parental over-protection was associated with a higher risk of PTSD in adulthood.  Protective factor: Parental over-protection | 1.Not having a controlling mother → lower risk of PTSD  OR(95%CI) = 4.84(2.26,10.3), *p* = 0.01  2.Having a caring father → lower risk of PTSD  OR(95%CI) =2.46 (1.18,5.12), *p* = 0.02  3.Not having controlling parents' → lower risk of PTSD  OR(95%CI) =2.70 (1.10,6.63), *p* = 0.04 |
| De Venter et al. (2013) | Family abuse | Depression and anxiety | N = 65articles  (Meet the standards) | Meta analysis | | ***Broadly yes:** 1. the occurrence of emotional, sexual and physical child abuse was the most important risk factor for the development of depression. 2. Sexual child abuse and family violence were greatest risk factors for anxiety disorders.  Risk factor: Family abuse | N/A |
| Weich et al. (2009) | Family abuse | 1. **Depression** 2. **Anxiety** 3. **PTSD** | N = 23 articles  (Meet the standards) | Narrativesynthesis | | ***Broadly yes:** Abusive relationships predicted depression, anxiety  and PTSD.  Risk factor: Family abuse | N/A |
| Johnson et al. (2002) | Childhood physical and sexual abuse  (New York Records;interview) | **Suicide attempt**  (Interview) | N = 659 families | Logistic regression | | ***Yes:** Childhood abuse significantly predicted offspring’s suicide attempts.  Risk factor: Family abuse (Physical and sexual abuse) | 1.Physical abuse → suicide attempt  OR(95%CI) = 5.10 (1.78,14.64)  2.Sexual abuse → suicide attempt  OR (95%CI) = 7.22 (2.22,23.53) |
| Tran et al. (2015) | Abuse (13 categories, such as emotional/physical/sexual abuse et al.)  (Adverse Childhood Experiences International Questionnaires, ACE-IQ) | Mental health  (Center for Epidemiological Studies-Depression scale, CES-D; anxiety scale developed in Vietnam;WHO-5 Well-being Index;Subjective Happiness Scale) | N = 2,099 | ANOVA | | ***Yes:**There were strong associations between the number of adverse childhood experiences and more severe depression, anxiety as well as lower well-being and happiness.  Risk factor: Family abuse | N/A |
| McLaughlin et al. (2011) | Child SES  (Parental education; financial hardship; parental occupation) | Onset, persistence and severity of mood, anxiety, behavior, substance disorders  (NCS-R diagnoses) | N = 5,692 | logistic regression | | ***Yes:**1.Parental education was associated with persistence of behavior disorders; 2. Parental education predicted disorder severity.  Risk factor: Low parent education. | 1. OR = 1.6 2. 2.OR = 1.6 - 2.6 |
| Sonego et al. (2013) | Parental education  (Self-Reported) | **Mental health**  (Parent-reported Strengths and Difficulties Questionnaire) | N = 5,635, 4-11 years old children | Univariate and multivariate analyses | | ***Yes:** There is a strong association between parental education and parent-reported child mental health.  Risk factor: Low parent education. | Ref: Both parents, university  1.For children: OR = 1.6 - 3.7  2.For adolescent: OR = 0.9 -1.3  (*p* = 0.045) |
| Karreman et al. (2008) | Coparenting  (16-item Revised Coparenting Scale) | Effortful control  (Children’s behavior questionnaire,CBQ) | N = 89 families | Regression | | ***Yes:** Effortful control was predicted from coparenting.  Protective factor: Coparenting | R² = 0.09, *p<*0.05 |
| Umemura et al. (2015) | Coparenting  (Coparenting behaviors during the triadic interaction; Dyadic levels of coparenting behaviors; Parents’ individual behaviors within coparenting contexts) | Symptoms of psychological problems in children  (TRF; Child Behavior Checklist, CBCL) | N = 108 families | Regression | | ***Weakly yes:**Cooperatingcoparenting marginally predicted children’s symptoms of affective disorders.  Protective factor: Coparenting (Cooperating) | N/A  Cooperating coparenting→ children’s symptoms of affective  B = -1.68, *p<*0.01 |
| Yan et al. (2018) | Coparenting quality  (12-item scale developed and used  by Stright and Bales, 2003) | 1. Dyadic adjustment 2. (Dyadic Adjustment Scale) 3. 2. Negative interaction 4. (Negative Interaction Scale) | N = 182 couples | APIM | | ***Yes:** Parent’s coparenting quality waslinked with both one’s own and partner’s perceived relationship quality.  Protective factor: Coparenting | N/A  1.Coparenting quality→dyadic adjustment:  B = 0.12 - 0.14  2.Coparenting quality →negative interaction:  B = -(0.06 - 0.09) |
| McAdams andVaillant (1982) | Intimacy motivation  (The Thematic Apperception Test, TAT) | **Psychosocial adjustment** | N = 57, middle-aged men, longitudinal data | Correlation | | ***Yes:** High intimacy motivation at age 30 was significantly associated with better adjustment 17 years later.  Protective factor: Parental warmth | r = 0.39, *p<*0.01 |
| Beckmann (2021) | **Parental warmth**  (In line with Amato, 1990) | Depression and anxiety  (German translation of the Patient Health Questionnaire-4, PHQ-4) | N = 7,423 | Linear mixed effects regression model | | ***Yes:** Parental warmth was related to decreased anxiety and depression.  Protective factor: Parental warmth | R² =0.144  B = -1.122, *p<*0.001 |
| Paine et al. (2021) | Warm adoptive parenting  (10-item questionnaire) | Child emotional and behavioral problems (CBCL) | N = 96 families | Multilevel growth analyses | | ***Yes:** Warm adoptive parenting was associated with a marked reduction in children's internalizing and externalizing problems over time.  Protective factor: Parental warmth | N/A   1. For internalizing problem 2. B = -5.36, *p<*0.01 3. 2.For externalizing problem 4. B = -7.18, *p<*0.01 |
|  |  |  |  |  | |  |  |
| **Parent-child relationship** | |  |  |  | |  |  |
| Stansfeld et al. (2008) | **Attachment** | Psychological distress  (Epidemiologic Studies Depression Scale, CES-D) | N = 7,276, civil servants from the Whitehall II Study. | Multinomial logistic regression | | ***Yes:** Depressive symptoms were associated with insecure attachment style.  Risk factor: Insecure attachment | OR = 1.44 - 3.13 |
| Kerstis et al. (2018) | Attachment to father and mother  (Relationship Structures (ECR-RS) questionnaire) | Depressive symptoms (DSM-IV) | N = 3,988 adolescents | Linear regression | | ***Yes:** Secure attachment was negatively associated with depression of offspring  Protective factor: Secure attachment | N/A   1. Mother → boys   B (95%CI) = -0.532(-0.656,0.407),  *P <*0.001   1. Mother → girls   B (95%CI) = -0.623(-0.730, 0.516),  *P<*0.001   1. Father → boys   B (95%CI) = -0.499(-0.608, 0.391),  *P<*0.001   1. Father → girls   B (95%CI) = -0.494(-0.586, 0.401),  *P<*0.001 |
| Tabachnick et al. (2022) | 1. Infant attachment quality 2. (Strange Situa-tion Procedure) 3. **2. Task type** 4. Positive or distress discussion | Emotion expression  (Video-recorded parent–child interaction task) | N = 78 families | ANOVA  regression analysis | | ***Yes:** 1. Secure attachmentwas associated with less positiveaffect in the distress discussion than insecure attachment but not in the positivediscussion. 2. Secure attachmentwas associated with less negative affect in the positive discussion than insecure attachment but not in the distress discussion.  Protective factor: Secure attachment | 1. F(1, 74) = 5.94, *p* = 0.02, η²_p_ = 0.07  2. F(1, 75) = 6.05, *p* = 0.02, η²_p_ = 0.08 |
| Sonuga-Barke et al. (2017) | **Deprivation**  (Who spent from soon after birth to up to 43 months in severe deprivation in Romanian institutions before being adopted.)  Group: ①deprivation *<* 6 months; ②deprivation > 6months; ③controls. | Interview  Symptoms of ASD, inattention and overactivity, disinhibited social engagement, conduct or emotional problems, and cognitive impairment. | N = 217, from the English and Romanian Adoptees study | Mixed-effects regression models | | ***Yes:** Extended early deprivation was associated with long-term deleterious effects.  Risk factor: Extended early deprivation | see Figure 2 in this article |
| McWey and Cui (2021) | 1. Contact with biological parents 2. (Youth reported the contact frequency) 3. **2.** Covariate: Maltreatment 4. (Four types: neglect, physical abuse, sexual abuse, and other) | Youth mental health symptoms  (Child Behavior Checklist,CBCL) | N = 247 → 101, longitudinal samples. | Multiple regression | | ***Partly Yes:**Frequency of contact with mother, rather than both parents, hada significant main effect on mental health symptoms.  Protective factor: Frequently contact with parents (mother) | Contact with biological mother →mental health symptom  B = −0.33, *p<*0.01 |
| Astle et al. (2020) | **Time with biological parents**  (by one question). | **Sexual behaviors**  (by asking) | N = 2,556 | Logistic regression | | ***Partly yes:** More time spent with biological parents predicted delayed a later onset of sexual behaviors. | 1. For vaginal sex   OR = 0.91, p <0.001   1. For oral sex   OR = 0.92, p < 0.001   1. For anal sex   OR = 0.95, p <0.01 |
| Cruz Fuentes et al. (2014) | Childhood psychosocial adversities  （Molecular analyses） | Depression  (World Mental Health Composite International Diagnostic Interview for Adolescents) | N = 516  Groups(n) = MDD (246); controls (270). | ANOVA, *χ*^2^ tests, logistic regression analysis | | ***Mostly yes:**Childhood psychosocial adversities have a significant impact on childhood depression.  Risk factor: Family abuse, Parental death | 1. Abuse, neglect, and family dysfunctions→clinical depression in adolescents:   For male:  OR(95%CI) = 2.9(1.8,4.5), *p<*0.05.  For female:  OR(95%CI) = 3.9(2.7,5.4), *p<*0.05.   1. Have experienced a life-threatening physical illness →clinical depression in adolescents 2. For male:   OR(95%CI) = 2.3(1.1,4.6), *p<*0.05  For female: OR(95%CI) = 2.2(1.1,4.6), *p<*0.05.   1. Parental death →clinical depression in adolescents   Only for female:  OR(95%CI) = 2.0(1.0,3.8), *p<*0.05. |
| Qin et al. (2022) | Alienation  (Inventory of Alienation Toward Parents) | Depression  (Childhood Depression Inventory) | N = 877 left-behind children, longitudinal data | Hierarchical Linear Model | | ***Yes:** Alienation toward parents at baseline predicted depression after one year.  Risk factor: Alienation with parents | B = 0.09, p < 0.01 |
| Susukida et al. (2016) | **Perceived love from caregivers in childhood**  (by asking) | **Lifetime suicidal ideation**  (by asking) | N = 5,692, from National Comorbidity Survey Replication, 2001-2003. | Multivariable logistic regression analyses | | ***Yes:** Perceived love from caregivers predicted lower lifetime suicidal ideation.  Protective factor: Perceived love from caregivers. | OR(95%CI) = 0.56 (0.38,0.82), *p<*0.01 |
| Koskenvuo and Koskenvuo (2015) | **Childhood adversities**  (by asking) | Use of psychotropic drugs  (National-Drug-Prescription-Register data) | N = 24,284 Finns, from the Health and Social Support | Logistic regression models; Multinomial regression models | | ***Yes:** There was a strong association between childhood adversities and psychotropic drug use .  Risk factor: Fear of family members | 1. Frequent fear of a family member → multiple use of antidepressants:   OR (95%CI) = 3.08 (2.72,3.49)   1. Frequent fear of a family member → multiple use of anxiolytics:   OR (95%CI) = 2.69 (2.27,3.20) |
| Tran et al. (2013) | Fear of family members  (Coincidental life adversity was assessed in a singlequestion) | Common mental disorders (CMD)  (Edinburgh Postnatal Depression Scale-Viet Nam Validation, EPDS) | N = 378 women | Equation Modeling analysis | | ***Yes:** Persistent CMD were predicted by fear of other family members.  Risk factor: Fear of family members | OR (95%CI) = 1.34 (1.13 - 1.6) |
| Note.*indicated supporting evidences for risk and protective factors in family of origin. | | | | | | | |
