# Supplementary material for: Family dominant hypothesis for the effect of family of origin on the mental health of offspring: evidence, mechanism, and implications
Source: Front Psychiatry. 2026 Jan 28;17:1733077. doi: 10.3389/fpsyt.2026.1733077 (PMC12891180; doi:10.3389/fpsyt.2026.1733077)
Supplement: Supplementary Figure 1 — Latent causal interactions among family variables based on Family Dominant Hypothesis. [file Table2.docx]

Supplementary material


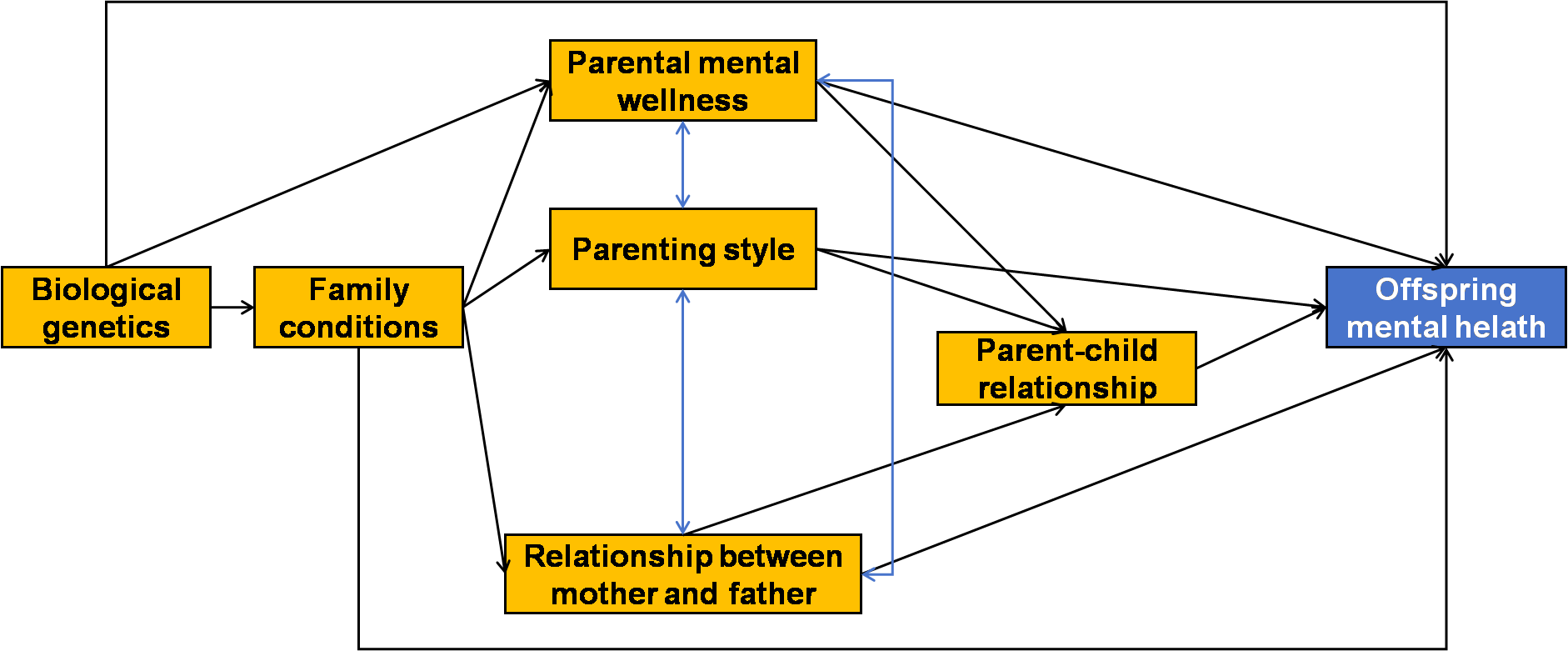


**Figure S1. Latent causal interactions within family of origin variables based on Family Dominant Hypothesis**
